# Veränderung des Bewegungsverhaltens durch die ersten beiden Coronawellen im Frühling und Winter 2020: Unterschied zwischen sportaffinen und nicht-sportaffinen Personen

**DOI:** 10.1007/s11553-022-00953-1

**Published:** 2022-05-31

**Authors:** Miriam Müller, Susanne Tittlbach

**Affiliations:** BaySpo – Bayreuther Zentrum für Sportwissenschaft, Universität Bayreuth, Universitätsstr. 30, 95447 Bayreuth, Deutschland

**Keywords:** COVID-19, Körperliche Aktivität, Bewegung, Psyche, Sportaffinität, COVID-19, Physical activity, Exercise, Mental health, Sports affinity

## Abstract

**Hintergrund:**

Da Bewegungsmangel weltweit zu den führenden Risikofaktoren für nicht-übertragbare Krankheiten zählt, ist von besonderem Interesse, wie sich die Coronapandemie auf das Bewegungsverhalten auswirkt.

**Fragestellung:**

Die vorliegende Studie untersucht die coronabedingte Veränderung der Bewegungsaktivitäten deutscher Erwachsener sowie Zusammenhänge der Aktivitätsveränderung mit der psychischen Gesundheit. Dabei wird ein Schwerpunkt auf Unterschiede zwischen sportaffinen und nicht-sportaffinen Personen gelegt.

**Methoden:**

Im Rahmen einer quantitativen Querschnittsstudie wurden Daten zum Aktivitätsausmaß 329 deutscher Erwachsener vor und während der ersten beiden Coronawellen mit Hilfe eines Online-Fragebogens erhoben.

**Ergebnisse:**

Die Coronapandemie führt im Durchschnitt zu einem signifikanten Rückgang des Aktivitätsausmaßes um 56,81 min/Woche. Diese Entwicklung ist allerdings nur bei sportaffinen Personen zu verzeichnen, wohingegen die nicht-sportaffine Gruppe ihr Aktivitätsausmaß um etwa 100 min/Woche erhöht. Ferner korreliert ein verringertes Bewegungsausmaß signifikant positiv mit einer schlechteren psychischen Gesundheit.

**Schlussfolgerung:**

Der coronabedingte Bewegungsrückgang verbunden mit einer schlechteren psychischen Gesundheit ist aufgrund möglicher langfristiger Folgen für die öffentliche Gesundheit besorgniserregend. Das gesteigerte Aktivitätsausmaß in der nicht-sportaffinen Gruppe stellt allerdings eine vielversprechende Entwicklung dar und sollte in Bezug auf künftige gesundheitsfördernde Maßnahmen berücksichtigt werden.

## Abstract (en)

**Background:**

As physical inactivity is one of the leading risk factors for noncommunicable diseases worldwide, it is of particular interest how the coronavirus pandemic affects physical activity behavior.

**Objectives:**

This study examines coronavirus-related changes in physical activity among German adults and associations of activity change with mental health. Emphasis will be placed on differences between sports enthusiasts and non-sports enthusiasts.

**Methods:**

As part of a quantitative cross-sectional study, data on the activity levels of 329 German adults were collected before and during the first two coronavirus waves using an online questionnaire.

**Results:**

On average, the coronavirus pandemic led to a significant decrease in the physical activity level by 56.81 min/week. However, this trend was only seen in sports enthusiasts, whereas the non-sports enthusiast group increased their activity level by approximately 100 min/week. Furthermore, reduced physical activity levels were significantly positively correlated with poorer mental health.

**Conclusions:**

The coronavirus-related decline in physical activity combined with poorer mental health is of concern because of potential long-term public health consequences. However, the increased activity level in the non-sports enthusiast group represents a promising development and should be considered in relation to future health promotion interventions.

## Einleitung

Die Coronapandemie und damit verbundene Schutzmaßnahmen^1^ haben seit 2020 drastische Auswirkungen auf das alltägliche gesellschaftliche Leben weltweit [14]. Bewegungsmangel zählt zu den führenden Risikofaktoren für nicht-übertragbare Krankheiten und verursacht hierzulande etwa 7,5 % aller Todesfälle [19]. Im Jahr 2018 bewegte sich mehr als jeder zweite deutsche Erwachsene zu wenig, womit Deutschland auf Platz 15 der inaktivsten Länder weltweit lag [12]. Es stellt sich die Frage, wie sich die coronabedingten Maßnahmen auf das Bewegungsverhalten der deutschen Bevölkerung auswirken und ob es unterschiedliche Entwicklungen zwischen sportaffinen und nicht-sportaffinen Personen gibt.

## Aktueller Forschungsstand

### Bewegungsausmaß

Während der ersten Coronawelle zeigt sich bei Erwachsenen weltweit eine Tendenz hin zu einem geringeren oder gleichbleibenden Bewegungsausmaß [8, 17, 20]. Gemessen an der Zahl derjenigen, die das von der WHO empfohlene Mindestmaß an Bewegung erreichen (150 min/Woche ausdauerorientierte Aktivitäten und an mindestens 2 Tagen/Woche muskelstärkende Aktivitäten), wird im Rahmen des „German COVID-19 Snapshot Monitoring“ (COSMO) im April 2020 jedoch nur ein leichter Rückgang von 22,6 % im Jahr 2014/15 auf 22,3 % festgestellt [5]. Eine italienische Studie kommt mit einem Rückgang von 34,9 % auf 24,6 % allerdings zu höheren Rückgängen. Zudem haben sich die transportbezogenen Aktivitäten durch die Coronapandemie mehr als halbiert und die durchschnittliche Zeit, die mit Sport und Bewegung in der Freizeit verbracht wird, sinkt ebenfalls signifikant um knapp 30 % [13]. Auch eine weltweite Online-Befragung belegt während der Coronapandemie in allen Bewegungsbereichen signifikant niedrigere Aktivitätswerte im Vergleich zu davor. Vor allem die Gehaktivitäten nehmen mit Verringerungen von bis zu 42 % stark ab. Allgemein sinkt das Bewegungsausmaß signifikant um 38 % [1].

### Unterschied zwischen sportaffinen und nicht-sportaffinen Personen

Unter sportaffin sind Personen zu verstehen, die vor der Coronapandemie das empfohlene Mindestmaß an Bewegung erreichen, die Bewegungsaktivitäten also einen hohen Stellenwert in ihrem Lebensstil zuweisen. Bezüglich unterschiedlicher Auswirkungen der Coronapandemie auf sportaffine und nicht-sportaffine Personen besteht in der aktuellen Forschungsdiskussion keine Einigkeit.

Während z. T. davon ausgegangen wird, dass zuvor aktive Personen ihr Bewegungsausmaß weiter erhöhen und inaktive sich weniger bewegen ([15]; sog. Schereneffekt), belegen andere Studien eine genau gegenteilige Entwicklung und weisen für sportaffine Personen eine Verringerung und für nicht-sportaffine Personen eine Erhöhung des Bewegungsausmaßes [6, 9] bzw. gleichbleibendes Aktivitätsausmaß [13, 16, 17] nach.

Andere Studien kommen zum Ergebnis, dass zuvor Inaktive ihre Bewegungsaktivitäten erhöhen und zuvor Aktive ihr Aktivitätsniveau beibehalten [7, 10]. Aufgrund der jeweiligen Anzahl an veröffentlichten Studien zeigt sich insgesamt eine Tendenz zu einer Bewegungsabnahme bei sportaffinen und einem gleichbleibendem oder leicht angestiegenen Bewegungsausmaß bei nicht-sportaffinen Personen.

### Psyche

In bislang publizierten Forschungsarbeiten zu Zusammenhängen zwischen dem Bewegungsverhalten und der psychischen Gesundheit während der Coronapandemie wird eine negative Veränderung des Aktivitätsausmaßes mit einem schlechteren positiven Wohlbefinden sowie mit einer erhöhten Auftrittswahrscheinlichkeit von Depressionen, Angst oder Stress assoziiert [2, 4, 16].

## Relevanz und Fragestellungen der Studie

Untersuchungen zu den Auswirkungen der Coronapandemie auf das Bewegungsverhalten in Deutschland sind bisher noch limitiert und es existieren nur wenige Forschungsarbeiten, die dabei den Einfluss der Sportaffinität berücksichtigen. Die vorliegende Studie untersucht daher die coronabedingte Veränderung des Bewegungsverhaltens deutscher Erwachsener und geht dabei schwerpunktmäßig auf mögliche Unterschiede zwischen sportaffinen und nicht-sportaffinen Personen ein. Da Infektionskrankheiten und Pandemien auch im sportwissenschaftlichen Kontext als eine der größten globalen Bedrohungen des 21. Jahrhunderts gelten, ist eine Analyse der Auswirkungen der Coronapandemie auf gesundheitliche Verhaltensweisen von sehr hoher Relevanz [18]. Die Ergebnisse können helfen, das Verhalten der Menschen in ähnlichen Situationen besser vorherzusagen und dadurch gezieltere gesundheitsfördernde Maßnahmen einzusetzen, um dem weltweit vorherrschenden Bewegungsmangel auch in Krisenzeiten entgegenwirken zu können. Konkret werden folgende Fragestellungen (FS) behandelt:

### FS 1.

In welchem Ausmaß hat sich das Bewegungsverhalten in den unterschiedlichen Aktivitätsdomänen durch die Coronapandemie verändert?

### FS 2.

Welche Zusammenhänge zeigen sich zwischen dem Aktivitätsausmaß und der psychischen Gesundheit während der Coronapandemie?

## Methodik

### Design

Die Feldstudie basiert auf einer quantitativen, querschnittlichen Datenerhebung eines „convenient samples“ in Form einer anonymen, internetgestützten Befragung. Die Datenerhebung wurde mit in Deutschland lebenden Erwachsenen vom 5. bis 25. Mai 2021 durchgeführt.

### Untersuchungsstichprobe

Die statistischen Analysen basieren auf einer Stichprobe von 329 Probanden (w: 63,5 %, m: 36,5 %; Altersverteilung: 18–39 Jahre [60,5 %], über 40 Jahre [39,5 %]; Schulabschluss: mindestens (Fach‑)Abitur [76,6 %]). 256 Probanden erfüllen mindestens eine der Bewegungsempfehlungen der WHO (mindestens 150 min/Woche ausdauerorientierte und/oder an mindestens 2 Tagen/Woche muskelkräftigende Aktivitäten) und gehören somit der sportaffinen Teilstichprobe an, wohingegen 68 Personen das Mindestmaß an Bewegung nicht erreichen und als nicht-sportaffin eingestuft werden. Die sportaffine Gruppe wird weiter untergliedert in AG_Ausdauer_ (= Erreichen ausdauerorientierte Bewegungsempfehlung, *n* = 95) und AG_beide_ (= Erreichen beide Bewegungsempfehlungen, *n* = 151).

### Erhebungsmethoden und Datenauswertung

Das Bewegungsausmaß vor und während der Coronapandemie wird mit dem European Health Interview Survey – Physical Activity Questionnaire (EHIS-PAQ) erfasst [11]. Um Zusammenhänge zwischen dem Aktivitätsausmaß und der psychischen Gesundheit während der Coronapandemie aufdecken zu können wird mit der Short Warwick-Edinburgh Mental Wellbeing Scale (SWEMWBS; [3]) die Höhe des allgemeinen mentalen Wohlbefindens während der Coronapandemie ermittelt sowie die Auftrittswahrscheinlichkeit der Gefühle Einsamkeit, Stress, Ängstlichkeit und Depression abgefragt.

Die Datenanalyse findet mittels χ^2^-Tests nach McNemar für nominal skalierte Merkmale sowie mittels Varianzanalysen mit Messwiederholung für intervallskalierte Variablen statt. Zur Überprüfung eines statistisch signifikanten Zusammenhangs zwischen der Veränderung des Bewegungsverhaltens und der psychischen Gesundheit wird der Korrelationskoeffizient nach Pearson bzw. der Eta- (η‑)Korrelationskoeffizient berechnet. Um Effekte der soziodemografischen Variablen zu kontrollieren, werden in allen Unterschiedsanalysen, Geschlecht, Alter und Bildung als Kovariate berücksichtigt.

## Darstellung der Ergebnisse

### Ausmaß

Bei einer allgemeinen Abfrage zur Veränderung des Aktivitätsausmaßes geben 50,5 % der Befragten an, sich durch die Coronapandemie weniger zu bewegen. Demgegenüber liegt der Anteil derer, die ihr Aktivitätsniveau beibehalten bzw. erhöhen bei je etwa einem Viertel (23,7 % bzw. 25,8 %). Diese Tendenz wird durch die detaillierte Auswertung des EHIS-PAQ-Fragebogens bestätigt (vgl. Tab. 1). So werden sowohl für transportbezogene Aktivitäten als auch für freizeitbezogene, ausdauerorientierte und muskelkräftigende Aktivitäten signifikante Rückgänge zwischen den beiden Zeiträumen nachgewiesen. Bei den gesamten ausdauerorientierten Aktivitäten (Radfahren und freizeitbezogene Aktivitäten) ist somit ein signifikanter Rückgang um 56,81 min/Woche zu konstatieren.

**Tab. 1:** Aktivitätsausmaß vor und während der Coronapandemie in den unterschiedlichen Aktivitätsdomänen

Aktivitätsdomänen	*n*	Vor der Coronapandemie (Mittelwert)	Während der Coronapandemie (Mittelwert)	Varianzanalyse mit Messwiederholung
*Transportbezogene Aktivitäten*				
Gehen (min/Woche)	329	**180,17**	**145,08**	F (1,328) = 11,42^a^; η^2^ = 0,03
Radfahren (min/Woche)	329	**75,81**	**50,91**	F (1,328) = 23,00^c^; η^2^ = 0,07
Gesamt (MET-min/Woche)	329	**1049,39**	**784,22**	F (1,328) = 25,77^c^; η^2^ = 0,07
*Freizeitbezogene Aktivitäten*				
Min/Woche	324	**276,81**	**244,58**	F (1;323) = 4,00^a^; η^2^ = 0,01
*Ausdauerorientierte Aktivitäten*				
Min/Woche	324	**350,31**	**293,50**	F (1,323) = 9,85^b^; η^2^ = 0,03
*Muskelkräftigungsübungen*				
Tage/Woche	329	**1,75**	**1,57**	F (1;328) = 3,91^a^; η^2^ = 0,01

^a^*p* < 0,05; ^b^*p* < 0,01; ^c^*p* < 0,001

Zudem können auch hinsichtlich der Erfüllung der Bewegungsempfehlungen der WHO signifikante Veränderungen durch die Coronapandemie dokumentiert werden. Die prozentualen Anteile derer, die mindestens 150 min/Woche aktiv sind (v: 75,9 %; w: 62 %), mindestens an 2 Tagen/Woche muskelkräftigende Übungen durchführen (v: 50,5 %; w: 40,4 %) oder beide Mindestempfehlungen erfüllen (v: 46,6 %; w: 35,5 %) sind durch die Coronapandemie signifikant mit mittlerer Effektstärke gesunken (χ^2^ [1] = 22,25***; V = 0,41; χ^2^ [1] = 9,39**; V = 0,35; χ^2^ [1] = 11,78**; V = 0,35 [***p < 0,001; **p < 0,01)]). Dem gegenüber steigt der Anteil derer, die keine der Mindestempfehlungen erreichen von 20,7 % auf 32,8 % signifikant an (χ^2^ [1] = 17,28***; V = 0,35 [***p < 0,001]).

### Unterschied sportaffin – nicht-sportaffin

**Abb. 1:**
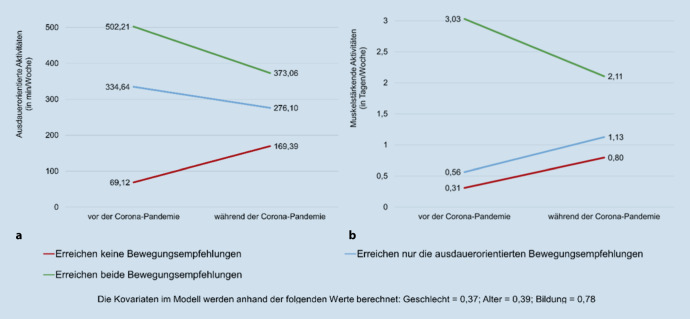
Graphische Darstellung der Bewegungsaktivitäten vor und während der Coronapandemie in unterschiedlichen Aktivitätsgruppen (*N* = 314; *n* AG_keine_ = 68; *n* AG_Ausdauer_ = 95; *n* AG_beide_ = 151): **a** ausdauerorientierte Aktivitäten (in min/Woche), **b** muskelstärkende Aktivitäten (in Tagen/Woche)

Wird zwischen den Aktivitätsgruppen AG_keine_, AG_Ausdauer_ und AG_beide_ unterschieden, zeigen sich sowohl für die ausdauerorientierten Aktivitäten (F [2,308] = 11,87***; η^2^ = 0,07) als auch für die muskelstärkenden Aktivitäten (F [2,308] = 33,03***; η^2^ = 0,18) signifikante Unterschiede bezüglich der coronabedingten Veränderung des Bewegungsverhaltens. Das Gesamtausmaß der aeroben Aktivitäten sinkt in der AG_beide_ bzw. AG_Ausdauer_ um etwa 2 h/Woche bzw. 1 h/Woche deutlich (Abb. 1a). Im Gegensatz dazu ist in der AG_keine_ – die zuvor mit einer durchschnittlichen Aktivitätszeit von knapp 70 min/Woche das Mindestmaß an Bewegung nicht erreicht – eine starke Erhöhung um 100 min/Woche zu verzeichnen, wodurch sie während der Coronapandemie mit etwa 170 min/Woche knapp über dem empfohlenen Mindestmaß liegt.

In Bezug auf die Tage, an denen muskelkräftigende Übungen durchgeführt werden, ist in der AG_beide_ eine signifikante Abnahme von etwa 3 auf 2 Tagen/Woche zu verzeichnen, während die beiden anderen Gruppen – die vor der Coronapandemie die Bewegungsempfehlungen von mindestens 2 Tagen/Woche Krafttraining nicht erreicht haben – während der Pandemie im Durchschnitt etwa einen halben Tag pro Woche häufiger muskelkräftigende Übungen machen (Abb. 1b). Die AG_beide_ ist zwar auch während der Coronapandemie die aktivste Gruppe bezüglich der ausdauerorientierten und muskelstärkenden Aktivitäten, jedoch verringert sich der Abstand zwischen den Gruppen durch die Coronapandemie.

### Psyche

Eine weitere wichtige Erkenntnis dieser Arbeit ist, dass die Veränderung des Bewegungsausmaßes in Krisensituationen wie der Coronapandemie ein entscheidender Faktor in Bezug auf die psychische Gesundheit zu sein scheint. Ein durch die Pandemie verringertes Bewegungsausmaß korreliert signifikant positiv mit einem schlechteren mentalen Wohlbefinden (|r| = 0,123*) sowie mit höheren Einsamkeits‑, Stress- und Depressionssymptomen (|r| = 0,131*; |r| = 0,113*; |r| = 0,137*). Mehr als jeder Zweite, der sein Bewegungsausmaß während der Coronapandemie verringert hat, gibt eine Verschlechterung des mentalen Wohlbefindens an, wohingegen nur 3,2 % dieser Gruppe eine bessere psychische Gesundheit aufweisen. Im Gegensatz dazu, berichtet in der Gruppe, die ihr Bewegungsausmaß durch die Coronapandemie erhöht hat, etwa jeder 4. (23,7 %) von einem verbesserten mentalen Wohlbefinden.

## Diskussion

### FS 1

Übereinstimmend mit bisherigen Forschungserkenntnissen [8, 17, 20] gibt auch in vorliegender Untersuchung etwa die Hälfte der Befragten an, sich durch die Coronapandemie weniger zu bewegen. Die Aktivitätswerte sind in vorliegender Befragung zwar während beider Zeiträume höher als in vorangegangenen Studien [13], jedoch ist eine vergleichbar hohe Abnahme des Bewegungsausmaßes in allen Aktivitätsdomänen zu verzeichnen. In Bezug auf die pandemiebedingte Veränderung des prozentualen Stichprobenanteils, der die Bewegungsempfehlungen erfüllt, kommt vorliegende mit einem Rückgang um etwa 10 % zu ähnlichen Ergebnissen wie bisherige Befragungen [13]. Das abweichende Ergebnis der COSMO-Studie im April 2020 [5] lässt vermuten, dass sich die Coronapandemie anfangs noch nicht so stark auf das Bewegungsverhalten ausgewirkt hat und erst nach einem längeren Lockdown signifikante Veränderungen zu erkennen sind.

Vorliegende Untersuchung zeigt eine Verringerung des aeroben Bewegungsausmaßes bei sportaffinen Personen und eine Erhöhung des Bewegungsausmaßes in der nicht-sportaffinen Gruppe und lässt sich damit neben folgenden Forschungsarbeiten einordnen [6, 9]. Die einzige deutsche Studie [17], die Unterschiede zwischen aktiven und inaktiven Personen untersucht, geht ebenfalls von einer Reduktion in der sportaffinen, allerdings von einem gleichbleibendem Bewegungsausmaß in der nicht-sportaffinen Gruppe aus.

### FS 2

Die Vermutung, dass die Veränderung des Bewegungsverhaltens mit dem psychischen Wohlbefinden korreliert, wird durch die Berechnung von Pearson-Korrelationen sowie durch die Erkenntnisse aktueller Studien [2, 4, 16] bestätigt. So ist eine Steigerung des aeroben Aktivitätsausmaßes signifikant mit einer besseren psychischen Gesundheit sowie mit geringeren Stress‑, Einsamkeits‑, Angst- und depressiven Symptomen verbunden. Da die AG_beide_ auch während der Coronapandemie noch die höchsten Aktivitätswerte, jedoch die schwächste psychische Gesundheit aufweist, wird vermutet, dass in Krisensituationen wie der Coronapandemie v. a. die Veränderung des Bewegungsverhaltens und nicht die Höhe des Aktivitätsausmaßes ein entscheidender Faktor in Bezug auf die psychische Gesundheit ist. Personen, die sich durch die Coronapandemie mehr bewegen, weisen unabhängig von der Dauer und Häufigkeit eine bessere mentale Gesundheit auf als Leute, bei denen das Bewegungsausmaß abnimmt. Allerdings muss in Bezug auf die Erkenntnisse zum Zusammenhang des Bewegungsverhaltens mit der psychischen Gesundheit beachtet werden, dass diese Korrelation beidseitig besteht. Es bleibt somit offen, ob die Höhe des Aktivitätsausmaßes das mentale Wohlbefinden beeinflusst oder der Zustand der psychischen Gesundheit das Bewegungsverhalten.

## Fazit für die Praxis

- Aufgrund des starken Bewegungsrückgangs durch die Coronapandemie sollten besonders in Krisenzeiten die positiven Aspekte von Bewegung herausgestellt und aktiv zu Sport und Bewegung aufgefordert werden.
- Da sich v. a. in Bezug auf die sportaffinen Gruppen negative Auswirkungen der Coronapandemie zeigen, sollten z. B. anhand von gemeinsamen Online-Trainings Alternativen gestaltet werden, die auch während eines Lockdowns den Spaß am Sport aufrechterhalten.
- Die nachgewiesene Korrelation der Veränderung des Bewegungsausmaßes mit psychischer Gesundheit verdeutlicht, dass Bewegungsförderung v. a. in psychisch herausfordernden Pandemiezeiten ein hilfreiches Mittel zur Stabilisierung des mentalen Wohlbefindens darstellt.
- Die Steigerung des Aktivitätsausmaßes in der nicht-sportaffinen Gruppe verdeutlicht, dass Krisensituationen auch positive Auswirkungen auf das Bewegungsverhalten erzielen können. Hier sollte versucht werden, diese Entwicklung auch nach der Coronapandemie beizubehalten.

## Footnotes

1 z. B. Ausgangs- und Kontaktbeschränkungen, Schließungen von Einzelhandel, Gastronomie, Fitnessstudios oder Schwimmbädern sowie abgesagte Trainings und Wettkämpfe.
